# Supplementary material for: Exploratory study to characterise the individual types of health literacy and beliefs and their associations with infection prevention behaviours amid the COVID-19 pandemic in Japan: a longitudinal study
Source: PeerJ. 2024 Feb 22;12:e16905. doi: 10.7717/peerj.16905 (PMC10894591; doi:10.7717/peerj.16905)
Supplement: Supplemental Information 2 [file peerj-12-16905-s002.docx]

Supplementary Table 1

1-a. Consideration for determining the number of clusters (in the case of four clusters).

|  | Cluster 1 (n = 310) | Cluster 2 (n = 2,110) | Cluster 3 (n = 1,945) | Cluster 4 (n = 1,635) |
| --- | --- | --- | --- | --- |
| General ease of infection | -1.833 | -0.544 | 0.205 | 0.806 |
| Extreme likelihood of infection | -1.569 | 0.013 | -0.303 | 0.641 |
| Low perception of infection threat | -0.703 | 0.590 | -0.427 | -0.120 |
| Ease of infection at dinners and parties | -1.828 | -0.235 | -0.176 | 0.859 |
| Ease of infection among young people | -1.598 | 0.040 | -0.337 | 0.653 |
| Excessive efficacy of infection control | -1.129 | 0.659 | -0.465 | -0.083 |
| Efficacy in vaccines and infection control | -1.852 | -0.096 | 0.008 | 0.465 |
| Dissatisfaction with the PCR testing system and vaccines | -1.287 | -0.182 | -0.082 | 0.576 |
| Inefficacy of therapeutic drugs | 0.481 | 0.079 | 0.023 | -0.220 |
| Hoax/conspiracy beliefs | -0.509 | 0.833 | -0.632 | -0.227 |
| Large social impact beliefs | -1.694 | -0.407 | 0.092 | 0.736 |
| China-originated beliefs | -0.885 | 0.186 | -0.510 | 0.534 |
| Optimism | -0.390 | 0.741 | -0.476 | -0.316 |
| Belief in just deserts | -0.866 | 0.358 | -0.545 | 0.351 |

1-b. Consideration for determining the number of clusters (in the case of five clusters).

|  | Cluster 1  (n = 1,773) | Cluster 2 (n = 1,425) | Cluster 3  (n = 228) | Cluster 4  (n = 1293) | Cluster 5 (n =1,281) |
| --- | --- | --- | --- | --- | --- |
| General ease of infection | 0.192 | 0.090 | -2.008 | 0.913 | -0.929 |
| Extreme likelihood of infection | -0.356 | 0.358 | -1.748 | 0.688 | -0.288 |
| Low perception of infection threat | -0.407 | 0.616 | -0.931 | -0.392 | 0.439 |
| Ease of infection at dinners and parties | -0.225 | 0.334 | -1.993 | 0.907 | -0.622 |
| Ease of infection among young people | -0.351 | 0.478 | -1.802 | 0.580 | -0.311 |
| Excessive efficacy of infection control | -0.450 | 0.748 | -1.389 | -0.357 | 0.399 |
| Efficacy in vaccines and infection control | 0.028 | 0.393 | -2.105 | 0.370 | -0.475 |
| Dissatisfaction with the PCR testing system and vaccines | -0.100 | 0.169 | -1.430 | 0.631 | -0.432 |
| Inefficacy of therapeutic drugs | 0.007 | -0.104 | 0.482 | -0.170 | 0.193 |
| Hoax/conspiracy beliefs | -0.641 | 0.740 | -0.666 | -0.456 | 0.643 |
| Large social impact beliefs | 0.066 | 0.063 | -1.850 | 0.881 | -0.722 |
| China-originated beliefs | -0.543 | 0.427 | -1.052 | 0.482 | -0.023 |
| Optimism | -0.460 | 0.645 | -0.626 | -0.623 | 0.659 |
| Belief in just deserts | -0.579 | 0.546 | -1.003 | 0.203 | 0.168 |

1-c. Consideration for determining the number of clusters (in the case of six clusters).

|  | Cluster 1  (n = 1,710) | Cluster 2  (n =190) | Cluster 3  (n = 1,063) | Cluster 4  (n = 1,122) | Cluster 5  (n = 1,062) | Cluster 6  (n = 853) |
| --- | --- | --- | --- | --- | --- | --- |
| General ease of infection | 0.232 | -2.307 | 0.163 | -0.998 | 0.959 | -0.034 |
| Extreme likelihood of infection | -0.113 | -1.904 | -0.413 | -0.311 | 0.804 | 0.572 |
| Low perception of infection threat | 0.216 | -0.832 | -0.807 | 0.436 | -0.463 | 0.760 |
| Ease of infection at dinners and parties | 0.058 | -2.228 | -0.301 | -0.672 | 0.991 | 0.405 |
| Ease of infection among young people | 0.176 | -1.856 | -0.647 | -0.350 | 0.618 | 0.559 |
| Excessive efficacy of infection control | 0.175 | -1.353 | -0.850 | 0.405 | -0.423 | 1.005 |
| Efficacy in vaccines and infection control | 0.374 | -2.248 | -0.250 | -0.535 | 0.340 | 0.342 |
| Dissatisfaction with the PCR testing system and vaccines | 0.021 | -1.533 | -0.144 | -0.469 | 0.698 | 0.226 |
| Inefficacy of therapeutic drugs | -0.239 | 0.447 | 0.204 | 0.227 | -0.133 | -0.008 |
| Hoax/conspiracy beliefs | -0.194 | -0.606 | -0.802 | 0.686 | -0.452 | 1.183 |
| Large social impact beliefs | 0.091 | -2.042 | 0.075 | -0.791 | 0.939 | 0.050 |
| China-originated beliefs | -0.106 | -1.053 | -0.666 | -0.037 | 0.562 | 0.625 |
| Optimism | 0.104 | -0.511 | -0.785 | 0.675 | -0.691 | 0.856 |
| Belief in just deserts | -0.213 | -1.016 | -0.676 | 0.180 | 0.310 | 0.873 |
