# Supplementary material for: Exploratory study to characterise the individual types of health literacy and beliefs and their associations with infection prevention behaviours amid the COVID-19 pandemic in Japan: a longitudinal study
Source: PeerJ. 2024 Feb 22;12:e16905. doi: 10.7717/peerj.16905 (PMC10894591; doi:10.7717/peerj.16905)
Supplement: Supplemental Information 3 [file peerj-12-16905-s003.docx]

Supplementary Table 2. Demographic profile in five clusters.

2-a. Cross table of sex×five clusters.

^＊^The numbers indicate the number of participants (%).

|  |  | Calm/hoax denial 1,773 (29.6) | Hoax affinity/threat denial 1,425 (23.8) | Minority/indifference 228 (3.8) | Over vigilance 1,293 (21.6) | Optimism 1,281 (21.4) | *p* value |
| --- | --- | --- | --- | --- | --- | --- | --- |
| Sex | Male 3,000 (50) | 911(15.2) | 659 (11.0) | 137 (2.3) | 601 (10.0) | 692 (11.5) | <0.001 |
|  | Female 3,000 (50) | 862 (14.4) | 766 (12.8) | 91(1.5) | 692 (11.5) | 589 (9.8) |  |

2-b. Cross table of marital status×five clusters

＊The numbers indicate the number of participants (%).

|  |  | Calm/hoax denial 1773 (29.6) | Hoax affinity/threat denial 1425 (23.8) | Minority/indifference 228 (3.8) | Over vigilance  1293 (21.6) | Optimism 1281 (21.4) | *p* value |
| --- | --- | --- | --- | --- | --- | --- | --- |
| Marital status | Unmarried 2,122 (35.4) | 667 (11.1) | 397 (6.6) | 143 (2.4) | 417 (7.0) | 498 (8.3) | <0.001 |
|  | Married (including separated or bereaved) 3,878 (64.6) | 1,106 (18.4) | 1,028 (17.1) | 85 (1.4) | 876 (14.6) | 783 (13.1) |  |

2-c. Cross table of the presence or absence of children×five clusters

＊The numbers indicate the number of participants (%).

|  |  | Calm/hoax denial 1773 (29.6) | Hoax affinity/ threat denial 1425 (23.8) | Minority/indifference 228 (3.8) | Over vigilance 1293 (21.6) | Optimism 1281 (21.4) | *p* value |
| --- | --- | --- | --- | --- | --- | --- | --- |
| The presence or absence of children | Having children living together 1,800 (30.0) | 501 (8.4) | 454 (7.6) | 49 (0.9) | 404 (6.8) | 392 (6.6) | <0.001 |
|  | Having children but not living together 1,319 (22.0) | 359 (6.0) | 411 (6.9) | 19 (0.4) | 290 (4.9) | 240 (4.0) |  |
|  | No children 2,881 (48.0) | 913 (15.3) | 560 (9.4) | 160 (2.7) | 599 (10) | 649 (10.9) |  |
